# 4,5-Dihydro­cyclo­penta­[*b*]thio­phen-6-one

**DOI:** 10.1107/S1600536811056042

**Published:** 2012-01-14

**Authors:** Lyall R. Hanton, Stephen C. Moratti, Zheng Shi, Jim Simpson

**Affiliations:** a Department of Chemistry, University of Otago, PO Box 56, Dunedin, New Zealand

## Abstract

The title compound, C_7_H_6_OS, crystallizes with two similar mol­ecules, 1 and 2, in the asymmetric unit. Both mol­ecules are essentially planar with r.m.s. deviations of 0.0193 and 0.0107 Å for the planes through the nine non-H atoms of mol­ecules 1 and 2, respectively. The thio­phene and 4,5-dihydro­cyclo­penta­dienone rings are inclined at 2.40 (13)° in 1 and 0.64 (13)° in 2. In the crystal structure π–π [3.6542 (17) Å] and C—H⋯π contacts stack the mol­ecules into columns in an inverse fashion along the *b* axis. An extensive series of C—H⋯O hydrogen bonds links the columns, generating an extended network structure.

## Related literature

For low band-gap and fluorescent applications of conjugated thio­phene vinyl­ene oligomers, see: Blanchard *et al.* (1997 ▶, 1998*a* ▶,*b* ▶, 2006 ▶). For control of the band-gap in the corres­ponding polymers, see: Roncali *et al.* (1994 ▶). For standard bond lengths, see: Allen *et al.* (1987 ▶). For related structures, see: Chang *et al.* (2004 ▶); Bonini *et al.* (2004 ▶). For the synthetic route to the starting material, methyl 6-oxo-5,6-dihydro-4*H*-cyclo­penta­[*b*]thio­phene-5-carboxyl­ate, see: Cai *et al.* (2002 ▶); More & Finney (2002 ▶); Yang (2009 ▶).
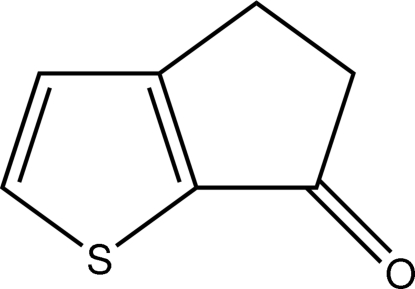


## Experimental

### 

#### Crystal data

C_7_H_6_OS
*M* *_r_* = 138.18
Triclinic, 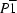

*a* = 6.6133 (9) Å
*b* = 7.4894 (11) Å
*c* = 13.3213 (16) Å
α = 83.247 (8)°
β = 86.097 (7)°
γ = 71.363 (8)°
*V* = 620.54 (14) Å^3^
*Z* = 4
Mo *K*α radiation
μ = 0.42 mm^−1^
*T* = 92 K
0.55 × 0.28 × 0.02 mm

#### Data collection

Bruker APEXII CCD area-detector diffractometer
Absorption correction: multi-scan (*SADABS*; Bruker, 2011 ▶) *T*_min_ = 0.617, *T*_max_ = 0.745
5251 measured reflections
1522 independent reflections
1264 reflections with *I* > 2σ(*I*)
*R*_int_ = 0.040
θ_max_ = 22.1°

#### Refinement

*R*[*F* ^2^ > 2σ(*F* ^2^)] = 0.040
*wR*(*F* ^2^) = 0.119
*S* = 1.11
1522 reflections
163 parameters
H-atom parameters constrained
Δρ_max_ = 0.30 e Å^−3^
Δρ_min_ = −0.34 e Å^−3^

### 

Data collection: *APEX2* (Bruker, 2011 ▶); cell refinement: *APEX2* (Bruker, 2011 ▶) and *SAINT* (Bruker, 2011 ▶); data reduction: *SAINT*; program(s) used to solve structure: *SHELXS97* (Sheldrick, 2008 ▶) and *TITAN2000* (Hunter & Simpson, 1999 ▶); program(s) used to refine structure: *SHELXL97* (Sheldrick, 2008 ▶) and *TITAN2000*; molecular graphics: *SHELXTL* (Sheldrick, 2008 ▶) and *Mercury* (Macrae *et al.*, 2008 ▶); software used to prepare material for publication: *SHELXL97*, *enCIFer* (Allen *et al.*, 2004 ▶), *PLATON* (Spek, 2009 ▶) and *publCIF* (Westrip, 2010 ▶).

## Supplementary Material

Crystal structure: contains datablock(s) global, I. DOI: 10.1107/S1600536811056042/tk5042sup1.cif

Structure factors: contains datablock(s) I. DOI: 10.1107/S1600536811056042/tk5042Isup2.hkl

Supplementary material file. DOI: 10.1107/S1600536811056042/tk5042Isup3.cml

Additional supplementary materials:  crystallographic information; 3D view; checkCIF report

## Figures and Tables

**Table 1:** Hydrogen-bond geometry (Å, °) *Cg*1 and *Cg*4 are the centroids of the S1,C12,C13,C17,C18 and S2,C22,C23,C27,C28 thio­phene rings, respectively.

*D*—H⋯*A*	*D*—H	H⋯*A*	*D*⋯*A*	*D*—H⋯*A*
C14—H14*A*⋯O1^i^	0.99	2.52	3.473 (4)	160
C15—H15*B*⋯O1^ii^	0.99	2.59	3.489 (3)	151
C12—H12⋯O2^iii^	0.95	2.43	3.370 (4)	168
C13—H13⋯O2^iv^	0.95	2.65	3.275 (4)	124
C23—H23⋯O1^v^	0.95	2.62	3.425 (4)	143
C24—H24*A*⋯O2^vi^	0.99	2.52	3.480 (4)	164
C25—H25*A*⋯O2^vii^	0.99	2.71	3.704 (3)	180
C25—H25*B*⋯*Cg*1^viii^	0.99	2.79	3.561 (3)	135
C15—H15*A*⋯*Cg*4^iii^	0.99	2.84	3.571 (3)	131

Symmetry codes: (i) 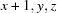; (ii) 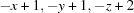; (iii) 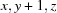; (iv) 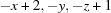; (v) 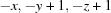; (vi) 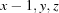; (vii) 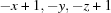; (viii) 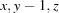.

**Fig. 1.**
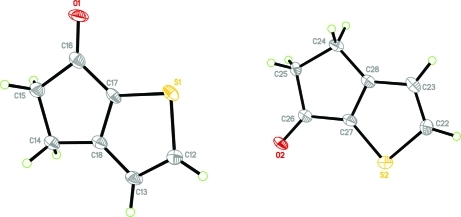
The asymmetric unit of I with ellipsoids drawn at the 50% probability level.

**Fig. 2.**
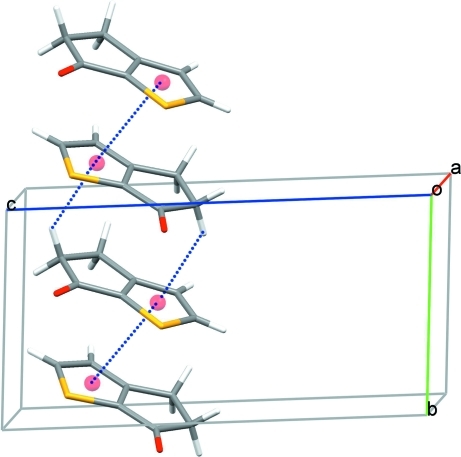
π–π and C–H···π contacts forming columns along b, with contacts shown as dashed lines.

**Fig. 3.**
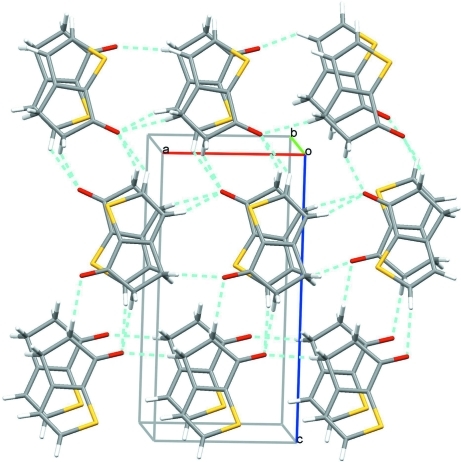
A general view of the crystal packing for the title compound with hydrogen bonds drawn as dashed lines.

## supplementary crystallographic information

### Comment

The title compound is a key intermediate in the synthesis of many conjugated thiophene vinylene oligomers for low band-gap and fluorescent applications (Blanchard *et al.*, 1997, 1998*a*,*b*, 2006). This is because the ethylene bridge in the resulting vinylene system flattens the ring and increases the conjugation between the neighbouring thiophene groups. This technique can also be used to control the band-gap in the corresponding polymers (Roncali *et al.*, 1994)

The asymmetric unit of the triclinic unit cell of the title compound, I, contains two unique molecules, 1 and 2, Fig 1. These are closely similar and overlay (Macrae *et al.*, 2008) with an r.m.s. deviation of only 0.024 Å. The molecules are approximately planar with r.m.s. deviations of 0.0193 and 0.0107 Å from the best fit planes through the nine non-hydrogen atoms in each of the two molecules, respectively. This is further illustrated by the fact that the thiophene and cyclopentadienone ring planes are inclined at 2.40 (13)° in molecule 1 and 0.64 (13)° in molecule 2. Bond distances in the molecule are normal (Allen *et al.*, 1987) and similar to those observed in related molecules (Chang *et al.*, 2004; Bonini *et al.*, 2004).

In the crystal structure, molecules 1 and 2 are linked by C15–H15A···π and C25–H25B···π contacts into dimers. π–π Contacts of 3.6542 (17) Å, between the centroids of the thiophene rings of molecules 1 and 2, link these dimers, forming columns along the *b* axis, Fig. 2. An extensive series of C–H···O hydrogen bonds involving the carbonyl O atoms from both molecules join the columns into an extended network, Fig 3. Interestingly the S atoms are not involved in any close intermolecular interactions.

### Experimental

Methyl 6-oxo-5,6-dihydro-4*H*-cyclopenta[*b*]thiophene-5-carboxylate was prepared by literature methods (Cai *et al.* 2002; More & Finney, 2002; Yang, 2009). Methyl 6-oxo-5,6-dihydro-4*H*-cyclopenta[*b*]thiophene-5-carboxylate (124 mg, 0.63 mmol) was dissolved in KOH (0.5 *M*) solution (H_2_O/EtOH, *v*/*v*=1) and refluxed for 4 h. The solution was cooled to room temperature, extracted with EtOAc (3× 20 ml), then the combined organic layers were washed with water (2× 50 ml) and dried over MgSO_4_. The solvent was evaporated in vacuo to yield transparent gold crystals (52.3 mg, 0.38 mmol) 60% yield. ^1^H NMR (δ p.p.m., CDCl_3_, 300 Hz): 7.890 (1*H*, d, J=3.6 Hz, C_4_H_2_S), 7.045 (1*H*, d, J=3.6 Hz, C_4_H_2_S), 3.049–2.974 (4*H*, m, CH_2_CH_2_).

### Refinement

All H-atoms were refined using a riding model with d(C—H) = 0.95 Å for aromatic–H and 0.99 Å for CH_2_–H atoms, and with *U*_iso_ = 1.2*U*_eq_ (C). Crystals were very weakly diffracting and data of reasonable intensity could not be obtained beyond θ = 22°. This also contributes to the relatively poor data/parameter ratio observed for this refinement.

### Crystal data

**Table:**

C_7_H_6_OS	*Z* = 4
*M**_r_* = 138.18	*F*(000) = 288
Triclinic, *P*1	*D*_x_ = 1.479 Mg m^−^^3^
Hall symbol: -P 1	Mo *K*α radiation, λ = 0.71073 Å
*a* = 6.6133 (9) Å	Cell parameters from 1413 reflections
*b* = 7.4894 (11) Å	θ = 3.1–22.0°
*c* = 13.3213 (16) Å	µ = 0.42 mm^−^^1^
α = 83.247 (8)°	*T* = 92 K
β = 86.097 (7)°	Rectangular plate, yellow
γ = 71.363 (8)°	0.55 × 0.28 × 0.02 mm
*V* = 620.54 (14) Å^3^	

### Data collection

**Table:**

Bruker APEXII CCD area-detector diffractometer	1522 independent reflections
Radiation source: fine-focus sealed tube	1264 reflections with *I* > 2σ(*I*)
graphite	*R*_int_ = 0.040
ω scans	θ_max_ = 22.1°, θ_min_ = 1.5°
Absorption correction: multi-scan (*SADABS*; Bruker, 2011)	*h* = −6→6
*T*_min_ = 0.617, *T*_max_ = 0.745	*k* = −7→7
5251 measured reflections	*l* = −14→14

### Refinement

**Table:**

Refinement on *F*^2^	Primary atom site location: structure-invariant direct methods
Least-squares matrix: full	Secondary atom site location: difference Fourier map
*R*[*F*^2^ > 2σ(*F*^2^)] = 0.040	Hydrogen site location: inferred from neighbouring sites
*wR*(*F*^2^) = 0.119	H-atom parameters constrained
*S* = 1.11	*w* = 1/[σ^2^(*F*_o_^2^) + (0.0713*P*)^2^ + 0.0126*P*] where *P* = (*F*_o_^2^ + 2*F*_c_^2^)/3
1522 reflections	(Δ/σ)_max_ < 0.001
163 parameters	Δρ_max_ = 0.30 e Å^−^^3^
0 restraints	Δρ_min_ = −0.34 e Å^−^^3^

### Special details

**Table:**

Geometry. All e.s.d.'s (except the e.s.d. in the dihedral angle between two l.s. planes) are estimated using the full covariance matrix. The cell e.s.d.'s are taken into account individually in the estimation of e.s.d.'s in distances, angles and torsion angles; correlations between e.s.d.'s in cell parameters are only used when they are defined by crystal symmetry. An approximate (isotropic) treatment of cell e.s.d.'s is used for estimating e.s.d.'s involving l.s. planes.
Refinement. Refinement of *F*^2^ against ALL reflections. The weighted *R*-factor *wR* and goodness of fit *S* are based on *F*^2^, conventional *R*-factors *R* are based on *F*, with *F* set to zero for negative *F*^2^. The threshold expression of *F*^2^ > σ(*F*^2^) is used only for calculating *R*-factors(gt) *etc*. and is not relevant to the choice of reflections for refinement. *R*-factors based on *F*^2^ are statistically about twice as large as those based on *F*, and *R*- factors based on ALL data will be even larger.

### Fractional atomic coordinates and isotropic or equivalent isotropic displacement parameters (Å^2^)

**Table:**

	*x*	*y*	*z*	*U*_iso_*/*U*_eq_	
S1	0.46716 (12)	0.59750 (11)	0.65016 (6)	0.0210 (3)	
C12	0.6994 (5)	0.5881 (4)	0.5819 (2)	0.0205 (8)	
H12	0.7031	0.6465	0.5148	0.025*	
C13	0.8795 (5)	0.4873 (4)	0.6343 (2)	0.0196 (8)	
H13	1.0213	0.4668	0.6078	0.024*	
C14	0.9491 (5)	0.3065 (4)	0.8221 (2)	0.0193 (8)	
H14A	1.0453	0.3705	0.8447	0.023*	
H14B	1.0345	0.1774	0.8064	0.023*	
C15	0.7725 (5)	0.2999 (4)	0.9031 (2)	0.0196 (8)	
H15A	0.7831	0.1670	0.9267	0.023*	
H15B	0.7872	0.3637	0.9619	0.023*	
C16	0.5581 (5)	0.4015 (4)	0.8544 (2)	0.0195 (8)	
O1	0.3814 (3)	0.4238 (3)	0.89458 (15)	0.0248 (6)	
C17	0.6113 (5)	0.4662 (4)	0.7517 (2)	0.0176 (8)	
C18	0.8269 (5)	0.4181 (4)	0.7325 (2)	0.0165 (7)	
S2	0.47328 (13)	0.09696 (11)	0.12918 (6)	0.0222 (3)	
C22	0.2153 (5)	0.2131 (4)	0.0920 (2)	0.0228 (8)	
H22	0.1814	0.2782	0.0266	0.027*	
C23	0.0655 (5)	0.2020 (4)	0.1654 (2)	0.0223 (8)	
H23	−0.0840	0.2574	0.1574	0.027*	
C24	0.0802 (5)	0.0432 (4)	0.3591 (2)	0.0198 (8)	
H24A	−0.0149	−0.0345	0.3558	0.024*	
H24B	0.0024	0.1568	0.3937	0.024*	
C25	0.2881 (5)	−0.0727 (4)	0.4135 (2)	0.0185 (8)	
H25A	0.3004	−0.0146	0.4749	0.022*	
H25B	0.2877	−0.2040	0.4339	0.022*	
C26	0.4739 (5)	−0.0725 (4)	0.3396 (2)	0.0170 (8)	
O2	0.6635 (3)	−0.1458 (3)	0.35756 (15)	0.0232 (6)	
C27	0.3776 (5)	0.0332 (4)	0.2462 (2)	0.0181 (8)	
C28	0.1598 (5)	0.0976 (4)	0.2551 (2)	0.0181 (8)	

### Atomic displacement parameters (Å^2^)

**Table:**

	*U*^11^	*U*^22^	*U*^33^	*U*^12^	*U*^13^	*U*^23^
S1	0.0114 (5)	0.0241 (6)	0.0242 (5)	−0.0009 (4)	−0.0045 (4)	−0.0007 (4)
C12	0.0189 (19)	0.0248 (19)	0.0193 (17)	−0.0099 (15)	−0.0005 (14)	0.0000 (14)
C13	0.0099 (18)	0.0216 (18)	0.0250 (19)	−0.0011 (14)	0.0014 (14)	−0.0048 (14)
C14	0.0125 (18)	0.0215 (19)	0.0229 (18)	−0.0037 (14)	−0.0018 (14)	−0.0020 (14)
C15	0.0166 (19)	0.0200 (18)	0.0207 (17)	−0.0040 (14)	−0.0017 (14)	−0.0011 (14)
C16	0.016 (2)	0.0171 (19)	0.0265 (18)	−0.0050 (15)	−0.0030 (16)	−0.0075 (14)
O1	0.0106 (14)	0.0311 (14)	0.0318 (13)	−0.0052 (10)	0.0044 (11)	−0.0061 (10)
C17	0.0100 (18)	0.0170 (18)	0.0236 (17)	−0.0007 (14)	−0.0049 (13)	−0.0014 (14)
C18	0.0117 (18)	0.0167 (17)	0.0217 (18)	−0.0041 (13)	0.0000 (13)	−0.0049 (14)
S2	0.0177 (6)	0.0271 (6)	0.0217 (5)	−0.0079 (4)	0.0016 (4)	−0.0014 (4)
C22	0.023 (2)	0.0224 (19)	0.0212 (18)	−0.0055 (15)	−0.0037 (15)	0.0044 (14)
C23	0.0154 (19)	0.0226 (19)	0.0283 (19)	−0.0038 (15)	−0.0057 (16)	−0.0032 (15)
C24	0.0124 (18)	0.0219 (18)	0.0239 (18)	−0.0038 (14)	−0.0001 (14)	−0.0025 (14)
C25	0.0167 (19)	0.0197 (18)	0.0176 (17)	−0.0037 (14)	−0.0044 (14)	0.0013 (14)
C26	0.015 (2)	0.0152 (17)	0.0217 (18)	−0.0043 (14)	−0.0002 (14)	−0.0057 (13)
O2	0.0094 (14)	0.0281 (14)	0.0284 (13)	−0.0010 (10)	−0.0032 (10)	−0.0003 (10)
C27	0.0125 (19)	0.0203 (19)	0.0222 (17)	−0.0049 (14)	0.0008 (14)	−0.0064 (14)
C28	0.0138 (18)	0.0189 (18)	0.0221 (18)	−0.0047 (14)	−0.0013 (14)	−0.0052 (14)

### Geometric parameters (Å, °)

**Table:**

S1—C12	1.716 (3)	S2—C27	1.716 (3)
S1—C17	1.723 (3)	S2—C22	1.726 (3)
C12—C13	1.375 (4)	C22—C23	1.359 (4)
C12—H12	0.9500	C22—H22	0.9500
C13—C18	1.412 (4)	C23—C28	1.413 (4)
C13—H13	0.9500	C23—H23	0.9500
C14—C18	1.497 (4)	C24—C28	1.506 (4)
C14—C15	1.543 (4)	C24—C25	1.547 (4)
C14—H14A	0.9900	C24—H24A	0.9900
C14—H14B	0.9900	C24—H24B	0.9900
C15—C16	1.526 (4)	C25—C26	1.522 (4)
C15—H15A	0.9900	C25—H25A	0.9900
C15—H15B	0.9900	C25—H25B	0.9900
C16—O1	1.221 (4)	C26—O2	1.225 (4)
C16—C17	1.456 (4)	C26—C27	1.461 (4)
C17—C18	1.368 (4)	C27—C28	1.366 (4)
			
C12—S1—C17	90.47 (15)	C27—S2—C22	89.93 (15)
C13—C12—S1	113.1 (2)	C23—C22—S2	113.3 (2)
C13—C12—H12	123.5	C23—C22—H22	123.3
S1—C12—H12	123.5	S2—C22—H22	123.3
C12—C13—C18	111.3 (3)	C22—C23—C28	111.6 (3)
C12—C13—H13	124.3	C22—C23—H23	124.2
C18—C13—H13	124.3	C28—C23—H23	124.2
C18—C14—C15	103.4 (2)	C28—C24—C25	103.1 (2)
C18—C14—H14A	111.1	C28—C24—H24A	111.1
C15—C14—H14A	111.1	C25—C24—H24A	111.1
C18—C14—H14B	111.1	C28—C24—H24B	111.1
C15—C14—H14B	111.1	C25—C24—H24B	111.1
H14A—C14—H14B	109.0	H24A—C24—H24B	109.1
C16—C15—C14	107.4 (2)	C26—C25—C24	107.4 (2)
C16—C15—H15A	110.2	C26—C25—H25A	110.2
C14—C15—H15A	110.2	C24—C25—H25A	110.2
C16—C15—H15B	110.2	C26—C25—H25B	110.2
C14—C15—H15B	110.2	C24—C25—H25B	110.2
H15A—C15—H15B	108.5	H25A—C25—H25B	108.5
O1—C16—C17	128.2 (3)	O2—C26—C27	128.6 (3)
O1—C16—C15	126.6 (3)	O2—C26—C25	125.8 (3)
C17—C16—C15	105.1 (3)	C27—C26—C25	105.6 (3)
C18—C17—C16	112.5 (3)	C28—C27—C26	112.0 (3)
C18—C17—S1	112.4 (2)	C28—C27—S2	112.9 (2)
C16—C17—S1	135.1 (2)	C26—C27—S2	135.1 (2)
C17—C18—C13	112.7 (3)	C27—C28—C23	112.3 (3)
C17—C18—C14	111.5 (3)	C27—C28—C24	111.8 (3)
C13—C18—C14	135.8 (3)	C23—C28—C24	135.8 (3)

### Hydrogen-bond geometry (Å, °)

**Table:**

Cg1 and Cg4 are the centroids of the S1,C12,C13,C17,C18 and S2,C22,C23,C27,C28 thiophene rings, respectively.

**Table:**

*D*—H···*A*	*D*—H	H···*A*	*D*···*A*	*D*—H···*A*
C14—H14A···O1^i^	0.99	2.52	3.473 (4)	160.
C15—H15B···O1^ii^	0.99	2.59	3.489 (3)	151.
C12—H12···O2^iii^	0.95	2.43	3.370 (4)	168.
C13—H13···O2^iv^	0.95	2.65	3.275 (4)	124.
C23—H23···O1^v^	0.95	2.62	3.425 (4)	143.
C24—H24A···O2^vi^	0.99	2.52	3.480 (4)	164.
C25—H25A···O2^vii^	0.99	2.71	3.704 (3)	180.
C25—H25B···Cg1^viii^	0.99	2.79	3.561 (3)	135
C15—H15A···Cg4^iii^	0.99	2.84	3.571 (3)	131

Symmetry codes: (i) *x*+1, *y*, *z*; (ii) −*x*+1, −*y*+1, −*z*+2; (iii) *x*, *y*+1, *z*; (iv) −*x*+2, −*y*, −*z*+1; (v) −*x*, −*y*+1, −*z*+1; (vi) *x*−1, *y*, *z*; (vii) −*x*+1, −*y*, −*z*+1; (viii) *x*, *y*−1, *z*.

## References

[bb1] Allen, F. H., Johnson, O., Shields, G. P., Smith, B. R. & Towler, M. (2004). *J. Appl. Cryst.* **37**, 335–338.

[bb2] Allen, F. H., Kennard, O., Watson, D. G., Brammer, L., Orpen, A. G. & Taylor, R. (1987). *J. Chem. Soc. Perkin Trans. 2*, pp. S1–19.

[bb3] Blanchard, P., Brisset, H., Riou, A., Hierle, R. & Roncali, J. (1998*a*). *J. Org. Chem.* **63**, 8310–8319.

[bb4] Blanchard, P., Brisset, H., Riou, A., Hierle, R. & Roncali, J. (1998*b*). *New J. Chem.* **22**, 547–549.

[bb5] Blanchard, P., Brisset, H., Riou, A., Illien, B., Hierle, R., Riou, A. & Roncali, J. (1997). *J. Org. Chem.* **62**, 2401–2408. 10.1021/jo9623447 11671573

[bb6] Blanchard, P., Verlhac, P., Michaux, L., Frere, P. & Roncali, J. (2006). *Chem. Eur. J.* **12**, 1244–1255. 10.1002/chem.200500853 16281319

[bb7] Bonini, B. F., Capito, E., Comes-Franchini, M., Ricci, A., Bottoni, A., Bernardi, F., Miscione, G. P., Giordano, L. & Cowley, A. R. (2004). *Eur. J. Org. Chem.* pp. 4442–4451.

[bb8] Bruker (2011). *APEX2*, *SAINT* and *SADABS* Bruker AXS Inc., Madison, Wisconsin, USA.

[bb9] Cai, J. X., Zhou, Z. H., Zhao, G. F. & Tang, C. C. (2002). *Org. Lett.* **26**, 4723–4725. 10.1021/ol027197f 12489971

[bb10] Chang, K.-J., Rayabarapu, D. K. & Cheng, C.-H. (2004). *J. Org. Chem.* **69**, 4781–4787. 10.1021/jo049506g 15230602

[bb11] Hunter, K. A. & Simpson, J. (1999). *TITAN2000* University of Otago, New Zealand.

[bb12] Macrae, C. F., Bruno, I. J., Chisholm, J. A., Edgington, P. R., McCabe, P., Pidcock, E., Rodriguez-Monge, L., Taylor, R., van de Streek, J. & Wood, P. A. (2008). *J. Appl. Cryst.* **41**, 466–470.

[bb13] More, J. D. & Finney, N. S. (2002). *Org. Lett.* **17**, 3001–3003. 10.1021/ol026427n 12182609

[bb14] Roncali, J., Thobie-Gautier, C., Elandaloussi, E.-H. & Frere, P. (1994). *J. Chem. Soc. Chem. Commun.* pp. 2249–2250.

[bb15] Sheldrick, G. M. (2008). *Acta Cryst.* A**64**, 112–122. 10.1107/S0108767307043930 18156677

[bb16] Spek, A. L. (2009). *Acta Cryst.* D**65**, 148–155. 10.1107/S090744490804362X PMC2631630 19171970

[bb17] Westrip, S. P. (2010). *J. Appl. Cryst.* **43**, 920–925.

[bb18] Yang, B. (2009). World patent WO2009158380

